# SGLT-2 inhibitors and in-stent restenosis-related events after acute myocardial infarction: an observational study in patients with type 2 diabetes

**DOI:** 10.1186/s12916-023-02781-2

**Published:** 2023-02-24

**Authors:** Raffaele Marfella, Celestino Sardu, Nunzia D’Onofrio, Carlo Fumagalli, Lucia Scisciola, Ferdinando Carlo Sasso, Mario Siniscalchi, Ludovica Vittoria Marfella, Davide D’Andrea, Fabio Minicucci, Giuseppe Signoriello, Arturo Cesaro, Maria Consiglia Trotta, Chiara Frigé, Francesco Prattichizzo, Maria Luisa Balestrieri, Antonio Ceriello, Paolo Calabrò, Ciro Mauro, Luca del Viscovo, Giuseppe Paolisso

**Affiliations:** 1 Department of Advanced Medical and Surgical Sciences, University of Campania “Luigi Vanvitelli”, Naples, Italy; 2 Mediterranea Cardiocentro, Piazza Miraglia, 2, 80138 Naples, Italy; 3 Department of Precision Medicine, the University of Campania “Luigi Vanvitelli”, Naples, Italy; 4 Department of Cardiology, Hospital Cardarelli, Naples, Italy; 5 Department of Mental Health and Public Medicine, Section of Statistic, the University of Campania “Luigi Vanvitelli”, Naples, Italy; 6 Division of Clinical Cardiology, A.O.R.N. Sant’Anna e San Sebastiano’, University of Campania “Luigi Vanvitelli”, Caserta, Italy; 7 Department of Experimental Medicine, University of Campania “Luigi Vanvitelli”, Naples, Italy; 8 IRCCS MultiMedica, Via Fantoli 16/15, 20138 Milan, Italy

**Keywords:** Restenosis, Type 2 diabetes, SGLT-2 inhibitors, Major adverse cardiovascular events, Glycemic control

## Abstract

**Background:**

No study evaluated the incidence of intra-stent restenosis (ISR)-related events in patients with type 2 diabetes (T2DM) and acute myocardial infarction (AMI) treated or not with sodium/glucose cotransporter 2 inhibitors (SGLT2i).

**Methods:**

We recruited 377 patients with T2DM and AMI undergoing percutaneous coronary intervention (PCI). Among them, 177 T2DM were treated with SGLT2 inhibitors before PCI. The primary outcome was major adverse cardiovascular events (MACE) defined as cardiac death, re-infarction, and heart failure related to ISR. In patients without ISR, minimal lumen area and minimal lumen diameter were assessed by coronary CT-angiography at 1-year follow-up.

**Results:**

Glycemic control was similar in SGLT2i-treated patients and never SGLT2i-users. The incidence of ISR-related MACE was higher in never SGLT2i-users compared with SGLT2i-treated patients, an effect independent of glycemic status (HR = 0.418, 95% CI = 0.241–0.725, *P* = 0.002) and observed also in the subgroup of patients with HbA1c < 7% (HR = 0.393, 95% CI = 0.157–0.984, *P* = 0.027). In patients without the event, the stent patency was greater in SGLT2i-treated patients compared with never SGLT2i-users at 1-year follow-up.

**Conclusions:**

SGLT2i treatment in T2DM is associated with a reduced incidence of ISR-related events, independently of glycemic control.

**Supplementary Information:**

The online version contains supplementary material available at 10.1186/s12916-023-02781-2.

## Background

Restenosis, defined as the re-narrowing of an arterial lumen after corrective vascular intervention like percutaneous intervention (PCI) and coronary artery bypass graft surgery, is an increasingly important issue in clinical practice [1]. Indeed, as the number of stent placements has risen to over 3 million annually worldwide, revascularization procedures have become much more common [2]. Patients with type 2 diabetes (T2DM) have an accelerated rate of late loss of lumen diameter and an increased incidence of intra-stent restenosis (ISR) [3, 4], with T2DM being an independent predictor of recurrent restenosis [5, 6]. Although several large clinical trials have convincingly demonstrated that sodium-glucose co-transporter2 inhibitors (SGLT2i) improve cardiovascular outcomes in both T2DM and non-DM patients [7] and atherosclerosis progression [8], no data are available investigating the effects of SGLT2i on intra-stent restenosis in patients with AMI treated with revascularization and the possible relationships with glycemic status. Thus, we evaluated whether SGLT2i therapy is associated with lower rates of ISR-related events independently of glycemic control in T2DM patients with acute myocardial infarction (AMI).

## Methods

### Study design and population

This was an observational, prospective study evaluating the association between SGLT2i therapy and ISR in T2DM patients with AMI (ST-segment-elevation myocardial infarction-STEMI- and NSTEMI patients). Patients underwent successful stent implantation according to ACC/AHA/SCAI Guideline for Coronary Artery Revascularization [2]. Diabetes was categorized according to the American Diabetes Association criteria [9]. Furthermore, patients answered a specific questionnaire about medicines used for diabetes treatment before the beginning of the study, the date of the beginning and end of therapy, the route of administration, and the duration of use. Information from the medicine inventory during the research and this specific questionnaire was used to classify patients as “never SGLT2i-users” and “current SGLT2i-users.” Never SGLT2i-users were patients who never received SGLT2i before AMI nor during follow-up. The current SGLT2i-users were patients with ongoing SGLT2i therapy without discontinuation for at least 6 months before AMI and continued SGLT2i therapy without discontinuation during the follow-up. All patients completed the 12-month clinical follow-up through face-to-face interviews, phone calls, or medical chart review. Patients with heart failure, impaired renal function (eGFR < 60 ml/min, estimated through the CKD-EPI equation), coronary bypass indications, absence of coronary lesions, and malignancies were excluded from the study. The investigation conforms to the principles outlined in the Declaration of Helsinki for using human tissue or patients. The Institutional Review Board approved the protocol.

### Percutaneous coronary intervention and medical treatment

Routine analyses were obtained on admission before coronary angiography and before full medical therapy was started. Before PCI, all patients were administered loading doses of aspirin 200–300 mg and clopidogrel 300–600 mg; alternatively, ticagrelor 180 mg or prasugrel 60 mg was administered. PCI was performed via the femoral or radial approach after an intravenous bolus dose of heparin (50–100 U/kg) to achieve an activated clotting time of > 250 s. DAPT (a combination of aspirin 100 mg/day with clopidogrel 75 mg/day or ticagrelor 90 mg twice daily or prasugrel 5–10 mg/day) was recommended for > 12 months for patients who underwent PCI. Triple antiplatelet therapy (TAPT: cilostazol 100 mg twice daily in addition to DAPT) was left to the discretion of the individual operators. To stabilize glycemic control in the emergency setting, all patients underwent continuous insulin infusion: the infusion lasted until a stable glycemic goal (140–180 mg/dl) for at least 24 h. After that glycemic goal was maintained for 24 h, the infusion was stopped, and subcutaneous insulin was initiated. After discharge from the hospital, all patients were managed and followed for 12 months after PCI, as outpatients, to maintain an HbA1c level at < 7%. Diagnostic coronary angiography and PCI were performed using standard guidelines [2]. A successful PCI was defined as residual stenosis of < 30% and more than grade 3 flow in Thrombolysis In Myocardial Infarction flow for the infarct-related artery (IRA) after the procedure.

### Coronary CT angiography

Coronary CT angiography (CCTA) was performed in all patients at 1-year follow-up. All CCTA was performed with a 320 lines-wide detector row scanner capable of whole-heart coverage in 1 beat (Aquilion ONE, Genesis Edition, Canon Medical Systems, Tokyo, Japan) and combining 0.17-mm spatial resolution, 0.275 s gantry rotation time, FIRST®, and AiCE® reconstruction algorithm. Interpolation algorithm was used to obtain 640 0.5 axial sections with 50% overlap. A *Z*-axis coverage of 16 cm was used for coronary CTA in all patients. CCTA was conducted according to the recommendations of the Society of Cardiovascular Computed Tomography [10]. All patients received a 40–55 ml bolus of Iomeprolo 400 (Iomeron® 400 mg/ml, Bracco, Milan, Italy) at an infusion rate of 5.5/6.5 ml/s followed by 50 ml of saline solution. The scan window was based on HR. A body mass index–adapted scanning protocol was used, as previously described [5]. Datasets of each coronary CCTA examination were transferred to an image-processing workstation (VitreaTM Advanced Visualization, Canon Medical Europe) and analyzed by 2 readers, both with > 10 years of clinical experience in coronary CTA performance and analysis, blinded to the clinical findings. For any disagreement in data analysis between the 2 readers, a consensus agreement was achieved. The visualizing coronary segments were classified as interpretable when of adequate, good, or excellent image quality (scores 2 to 4). ISR > 50% were considered significant when assessing anatomy by CCTA. The location and extent of the region of interest were manually defined using proximal and distal markers as the coronary vessel region where the lumen diameter was reduced by ≥ 30% compared with the normal vessel. Planimetry of the inner lumen and outer vessel areas was performed following a stepwise approach. In summary, a centerline originating from the ostium was first automatically extracted and successively reacquired also manually to avoid potential misregistration errors; then, straightened and stretched multiplanar reformatted images were generated, and the lumen and vessel borders were detected longitudinally on 24 different vessel views by the software; based on these longitudinal contours, cross-sectional images at 0.25 mm intervals were calculated to create transversal lumen and vessel wall contours, which were examined and, if necessary, adjusted by a single experienced observer. Based on the detected contours proximal and distal from the lesion region, a reference area function was derived modeling the tapering of a healthy vessel. From these data, the following cross-sectional CTCA-derived parameters were automatically provided by operators: minimum lumen area (MLA), and % area stenosis (%AS) at the level of the MLA defined by [1-MLA/corresponding reference lumen area) × 100]. Minimum lumen diameter (MLD), less accurate parameter, was not considered.

### Clinical outcomes

The primary outcome was major adverse cardiovascular events (MACE) defined as cardiac death, acute coronary syndrome, and development of heart failure related to ISR. The relationship between cardiovascular events and restenosis was attributed by coronary angiography without evidence of other culprit lesions.

### Statistical analysis

For continuous variables, differences between the two groups were evaluated with the unpaired *t*-test. Data were expressed as mean ± standard deviation. For categorical variables, intergroup differences were analyzed using the chi-squared test or Fisher’s exact test, as appropriate. The Cox proportional hazard regression models, adjusted for admission HbA1c, 1-year HbA1c levels, age, hypertension, cigarette smoking, BMI, HDL, LDL-cholesterol levels, triglycerides, antithrombotic, and lipid-lowering and glucose-lowering therapies, were used to estimate the outcome. To reduce concerns that glycemic control during the study influenced results, a sensitivity analysis was conducted repeating the same analysis including only patients with 1-year mean HbA1c < 7% during the study period. All calculations were performed using the Data Analysis Software SPSS (IBM; Armonk, NY, USA).

## Results

### Baseline characteristics of the patients on admission at emergency wards

**Fig. 1:**
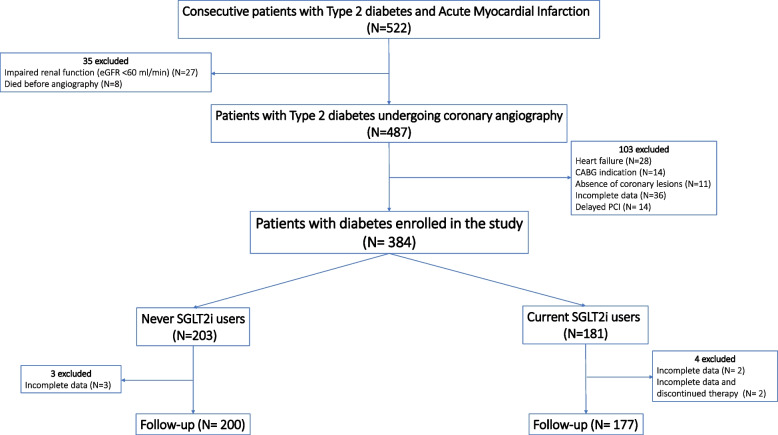
STROBE diagram

Among 522 T2DM patients with AMI who underwent successful stent implantation from November 2017 to March 2022, 377 were eligible for the study and had complete data at 12 months follow-up (Fig. 1). All patients underwent primary PCI within 3 h. Among the 377 patients enrolled in the study, 200 were never SGLT2i-users, and 177 were current SGLT2i-users. The duration of SGLT2i treatment was 18 ± 7 months (mean ± SD). During follow-up, there were no changes in the dose of SGLT2i. Twenty-one patients among never SGLT2i-users (10.5%) and 18 patients among current SGLT2i-users (10.2%) were treated with TAPT (*P* = 0.167). There were no differences in the mean age, BMI, sex distribution, smoking habits, HbA1c, admission glucose level, plasma cholesterol, and triglyceride levels among the groups. Angiographic data indicated that the treated lesion types were similar in the two groups. The stent type was also comparable between the two groups (Table 1). Using a mean of 2.2 matched angiographic projections per lesion, the reference diameter of the target vessel, the mean minimal luminal diameter (MLD), and the mean length of the lesion at baseline were similar in the two groups. After PCI, the MLD increased similarly in both groups. Moreover, the post-PCI stenosis was similar in both groups (Table 1).

**Table 1:** Baseline characteristics of the patients enrolled in the study

**Variable**	**SGLT-2i treated patients (*n* = 177)**	**Never SGLT-2i users (*n* = 200)**	***p-value***
Age (years)	66.2 ± 6.3	65.4 ± 6.1	0.416
BMI (kg/m^2^)	28.2 ± 2.2	28.0 ± 1.8	0.887
Male, *n* (%)	115 (65.0)	128 (64)	0.465
STEMI, *n* (%)	99 (55.9)	111 (55.5)	0.245
Diabetes duration, (years)	15.4 ± 3.2	14.9 ± 3.4	0.224
Hypertension, *n* (%)	97 (54.8)	110 (55.0)	0.526
Dyslipidemia, *n* (%)	70 (39.5)	73 (36.5)	0.595
Previous CVD, *n* (%)	58 (32.8)	63 (31.5)	0.439
Smokers, *n* (%)	29 (16.4)	33 (16.5)	0.544
Retinopathy, *n* (%)	30 (16.9)	34 (17.0)	0.437
Neuropathy, *n* (%)	37 (20.9)	41 (20.5)	0.511
**Admission therapy**			
Metformin, *n* (%)	106 (59.5)	119 (59.9)	0.512
DPP-IV inhibitors, *n* (%)	44 (24.9)	50 (25.0)	0.535
GLP-1 agonists, *n* (%)	19 (10.7)	22 (11.0)	0.504
Sulfonylureas, *n* (%)	37 (20.9)	42 (21.0)	0.442
Thiazolidinediones, *n* (%)	8 (4.5)	10 (5.0)	0.511
Insulin, *n* (%)	53 (29.9)	61 (30.5)	0.451
Multiple glucose-lowering drugs, *n* (%)	162 (91.5)	184 (92.0)	0.507
ACE inhibitors, *n* (%)	53 (29.9)	57 (28.5)	0.254
ARBs, *n* (%)	36 (20.3)	38 (19.0)	0.421
Beta-blockers, *n* (%)	62 (35.0)	74 (37.0)	0.386
Diuretics, *n* (%)	18 (10.2)	21 (10.5)	0.167
Calcium channel blockers, *n* (%)	10 (5.6)	12 (6.0)	0.305
Antiplatelet drugs, *n* (%)	117 (66.7)	134 (67.0)	0.466
Heparin, *n* (%)	6 (3.4)	8 (4.0)	0.397
Statin, *n* (%)	120 (67.8)	139 (69.5)	0.336
Systolic blood pressure (mmHg)	124.1 ± 8.6	124.0 ± 12.2	0.885
Diastolic blood pressure (mmHg)	78.6 ± 6.3	78.3 ± 6.3	0.587
Heart rate	83.5 ± 8.9	84.6 ± 10.5	0.587
Hba1c baseline	6.5 ± 1.6	6.3 ± 1.7	0.295
HbA1c 3 months	6.6 ± 0.7	6.6 ± 0.7	0.29
HbA1c 6 months	6.6 ± 0.7	6.6 ± 0.7	0.17
HbA1c 9 months	8.2 ± 1.2	8.1 ± 1.3	0.168
HbA1c 12 months	6.8 ± 0.7	6.9 ± 1.3	0.987
HbA1c 1-year mean	6.8 ± 0.5	6.9 ± 0.6	0.987
Total cholesterol (mg/dL)	179.6 ± 22.4	182.7 ± 15.3	0.764
LDL cholesterol (mg/dL)	104.5 ± 31.1	106.9 ± 18.1	0.763
HDL cholesterol (mg/dL)	39.9 ± 3.9	39.5 ± 3.5	0.596
Triglycerides (mg/dL)	183.4 ± 20.8	181.6 ± 24.1	0.594
Creatinine (mg/dL)	1.0 ± 0.1	1.0 ± 0.2	0.65
CK-MB (IU/L)	128.0 ± 7.9	127.9 ± 9.2	0.638
Troponin (ng/L)	37.4 ± 3.5	37.7 ± 3.6	0.129
NT-proBNP (pg/mL)	20696.0 ± 2119.7	20605.0 ± 2370.2	0.125
CRP (mg/dL)	14.8 ± 1.4	14.7 ± 1.3	0.124
Glucose (mg/dL)	150.6 ± 54.0	157.0 ± 51.7	0.133
Stenosis (%)	69.3 ± 5.9	69.8 ± 6.0	0.353
MLD (mm)	1.1 ± 0.2	1.1 ± 0.1	0.282
Post-stent MLD (mm)	2.5 ± 0.4	2.5 ± 0.3	0.441
Lesion length	21.4 ± 1.9	21.3 ± 2.1	0.368
Ref diameter	2.8 ± 0.4	2.8 ± 0.2	0.279

Data are presented as mean ± SD

*BMI* Body mass index, *HbA1c* Glycated hemoglobin, *CK-MB* Creatine phosphokinase-MB, *CRP* C-reactive protein, *MLD* Minimum lumen diameter

### Follow-up evaluation and CCTA analysis at 12 months from the PCI

**Fig. 2:**
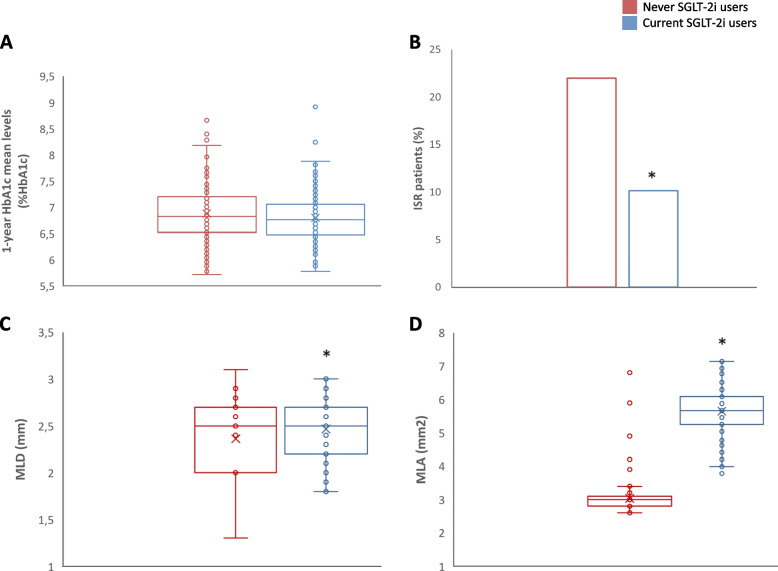
**A** 1-year HbA1c mean levels in never SGLT2i users (*n* = 200) and current SGLT2i users (*N* = 177), boxplots display the median, 25th, and 75th percentiles, range, and extreme values. **B** Intra-stent restenosis rate during 1-year follow-up in never SGLT2i users (*n* = 200) and current SGLT2i users (*N* = 177), *P < 0.05 vs never SGLT2i users. **C**,** D** Minimum lumen diameter (MLD) and minimal luminal area (MLA) of treated coronary by coronary CT angiography at 1-year follow-up in never SGLT2i users (*n* = 156) and current SGLT2i users (*N* = 159) without intra-stent restenosis-related events, *P < 0.05 vs never SGLT2i users. Boxplots display the median, 25th, and 75th percentiles, range, and extreme values

**Fig. 3:**
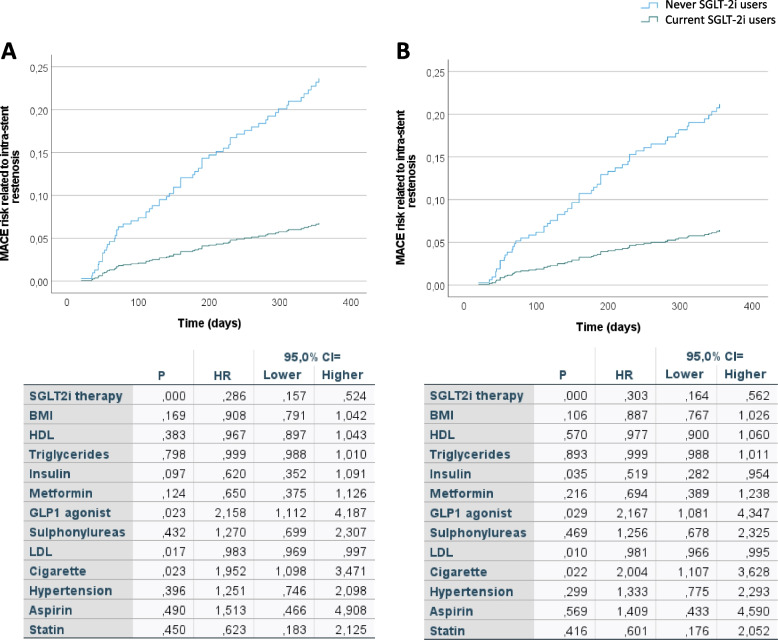
**A** Time-dependent MACE risk analysis, adjusted for admission HbA1c, 1-year HbA1c levels, age, hypertension, cigarette smoking, BMI, HDL, LDL-cholesterol levels, triglycerides, antithrombotic, and lipid-lowering and glucose-lowering therapies according to Cox regression analysis, along with the relative HR, 95% confidence interval, and *P* values for all the covariates used for the adjustment (table). **B** Time-dependent MACE risk analysis, adjusted for admission, age, hypertension, cigarette smoking, BMI, HDL LDL-cholesterol levels, triglycerides, antithrombotic, and lipid-lowering and glucose-lowering therapies according to Cox regression analysis, in never SGLT2i users (*n* = 118, 59%) and current SGLT2i users (*N* = 107, 60%) with good glycemic control (1-year HbA1c mean < 7%), along with the relative HR, 95% confidence interval, and *P* values for all the covariates used for the adjustment (table)

**Fig. 4:**
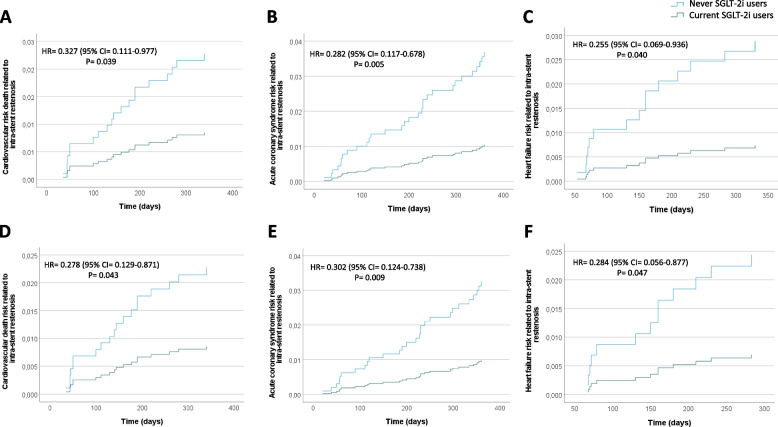
**A** Time-dependent cardiovascular death risk analysis, adjusted for admission HbA1c, 1-year HbA1c levels, age, hypertension, cigarette smoking, BMI, HDL, LDL-cholesterol levels, triglycerides, antithrombotic, and lipid-lowering and glucose-lowering therapies according to Cox regression analysis. **B** Time-dependent acute coronary syndrome risk analysis, adjusted for admission HbA1c, 1-year HbA1c levels, age, hypertension, cigarette smoking, BMI, HDL, LDL-cholesterol levels, triglycerides, antithrombotic, and lipid-lowering and glucose-lowering therapies according to Cox regression analysis. **C** Time-dependent heart failure risk analysis, adjusted for admission HbA1c, 1-year HbA1c levels, age, hypertension, cigarette smoking, BMI, HDL, LDL-cholesterol levels, triglycerides, antithrombotic, and lipid-lowering and glucose-lowering therapies according to Cox regression analysis. **D** Time-dependent cardiovascular death risk analysis, adjusted for age, hypertension, cigarette smoking, BMI, HDL, LDL-cholesterol levels, triglycerides, antithrombotic, and lipid-lowering and glucose-lowering therapies according to Cox regression analysis in never SGLT2i users (*n* = 118, 59%) and current SGLT2i users (*N* = 107, 60%) with good glycemic control (1-year HbA1c mean < 7%). **E** Time-dependent acute coronary syndrome risk analysis, adjusted for age, hypertension, cigarette smoking, BMI, HDL, LDL-cholesterol levels, triglycerides, antithrombotic, and lipid-lowering and glucose-lowering therapies according to Cox regression analysis in never SGLT2i users (*n* = 118, 59%) and current SGLT2i users (*N* = 107, 60%) with good glycemic control (1-year HbA1c mean < 7%). **F** Time-dependent heart failure risk analysis, adjusted for age, hypertension, cigarette smoking, BMI, HDL, LDL-cholesterol levels, triglycerides, antithrombotic, and lipid-lowering and glucose-lowering therapies according to Cox regression analysis in never SGLT2i users (*n* = 118, 59%) and current SGLT2i users (*N* = 107, 60%) with good glycemic control (1-year HbA1c mean < 7%)

There were no differences in the 1-year HbA1c mean levels among the groups (Fig. 2A). The incidence of ISR-related MACE was significantly higher in never SGLT2i-users (22.1%, *n* = 44) compared with current SGLT2i-users (10.2%, *n* = 18; *P* < 0.001) (Fig. 2B). The CT angiography, performed at follow-up in patients without ISR-related events, evidenced that the lesions treated with the stents deteriorated, resulting in a smaller MLA and MLD at follow-up in never SGLT2i-user compared with current SGLT2i-user (Fig. 2C, D). Multiple logistic regression was then performed to evaluate the relationship between the use of SGLT-2i and ISR-related MACE, adjusted for glycemic control (admission HbA1c and 1-year HbA1c mean), age, BMI, and sex. The probability of ISR-related MACE in patients treated with SGLT2i was reduced by 58 % regardless of glycemic control, age, BMI, and sex (OR, 0.42; 95% CI, 0.23–0.76). After 1 year following AMI, a multivariable Cox regression analysis, adjusted for admission HbA1c, 1-year HbA1c levels, age, hypertension, cigarette smoking, BMI, HDL, LDL-cholesterol levels, triglycerides, antithrombotic, and lipid-lowering and glucose-lowering therapies, showed a significantly higher 1-year risk of ISR-related events in never-SGLT2i-users compared to current-SGLT2i-users (Fig. 3A). In addition, to evaluate whether SGLT2i therapy was effective in patients with good glycemic control (1-year HbA1c mean < 7%), we performed a Cox regression analysis in this subgroup of patients, adjusted for admission age, hypertension, cigarette smoking, BMI, HDL, LDL-cholesterol levels, triglycerides, antithrombotic, and lipid-lowering and glucose-lowering therapies. The Cox analysis showed that also in patients with good glycemic control, SGLT2i therapy significantly reduced the risk of ISR-related MACE (Fig. 3B). After 1 year following AMI, a multivariable Cox regression analysis, adjusted for admission HbA1c, 1-year HbA1c levels, age, hypertension, cigarette smoking, BMI, HDL, LDL-cholesterol levels, triglycerides, antithrombotic, and lipid-lowering and glucose-lowering therapies, showed a significantly higher 1-year risk of ISR-related cardiovascular death (Fig. 4A), acute coronary syndrome (Fig. 4B), and heart failure (Fig. 4C) in never-SGLT2i-users compared to current-SGLT2i-users. The same trend for all the three outcomes considered was observed in the subgroup of patients with good glycemic control (1-year HbA1c mean < 7%) (Fig. 4D–F).

Subgroup analysis exploring the effect of single SGLT2i on the main composite outcome revealed there were no differences in the risk of ISR-related MACE among users of empagliflozin, canagliflozin, or dapagliflozin, while each of these groups has a significant lower rate compared with never-SGLT2i-users (Supplementary Fig. 1).

## Discussion

In this prospective observational study, we have evidence that SGLT2i therapy is associated with a reduction in the incidence of ISR-related MACE. Moreover, the effect of SGLT2i treatment on ISR outcomes was independent of the glycated hemoglobin levels at admission and during 1-year follow-up, thus suggesting that the benefit observed with SGLT2 inhibition on coronary reperfusion might be independent of their glucose-lowering properties.

No study has investigated the effect of SGLT2 inhibition in coronary stent outcomes after AMI. Despite advances in interventional skills, devices, and antiplatelet agents, outcomes of coronary revascularization in patients with diabetes have been poorer than in those without this condition. PCI in patients with diabetes is associated with an increased incidence of restenosis, repeated revascularization, and stent thrombosis than in those without diabetes [11]. In this context, for the first time, our data evidenced a reduction in ISR-related events in patients with T2DM and acute coronary syndrome treated with SGLT2i. Interestingly, the SGLT2i results on ISR were independent of glycemic control suggesting that SGLT2 inhibitors might favorably affect the coronary remodeling after stent implantation, possibly through the regulation of a large range of metabolic, molecular, and hemodynamic mechanisms irrespectively of their glucose-lowering effects [8, 12, 13]. Indeed, previous studies showed that in patients with T2DM and AMI, SGLT2i reduced infarct size and peri-infarct tissue inflammation [14], preventing also the loss of pump function expressed as LVEF [15]. These data might suggest that the anti-inflammatory effect of SGLT2i eventually contribute to their putative protective effect against restenosis. Consistently, our data suggest also that the protective effect of SGLT2i on ISR-related MACE is maintained in the subgroup of patients with good glycemic control. Of note, trials data demonstrated that SGLT2i reduce the risk of worsening heart failure or cardiovascular death in participants with heart failure, irrespective of diabetes status and glycemic control [7].

We also observed an improved stent patency in patients treated with SGLT2i. Among other factors, an inflammatory response driving immune cell infiltration and the subsequent proliferation of smooth muscle cells are held to underlie the risk of stent failure and in-stent restenosis [16]. The emerging anti-inflammatory activity of SGLT2i, both drug intrinsic and secondary to other mediators [17, 18], might eventually sustain the argument that these drugs limit local proliferation and the resulting thickening in the site of stent implantation, albeit this hypothesis must be validated by experimental data.

### Limitations of the study

Residual confounders are inherently linked to all observational studies. Despite our effort to control for all the most relevant risk factors, we did not monitor the dietary habits nor the amount of physical activity performed by the patients, two aspects that might have influenced the results. In addition, the comparator drugs were not a specific class, thus we could not perform a possible head-to-head comparison with drugs having known cardioprotective benefit, e.g., the GLP-1 receptor agonists [19]. In addition, the study was adequately powered to detect differences among different SGLT2i, albeit current literature suggests a generally homogenous class effect for these drugs [20].

## Conclusions

In summary, the use of SGLT2i is associated with a reduced incidence of cardiovascular events related to ISR in patients with diabetes. Intriguingly, the SGLT2i protective effects extend beyond glycemic control, suggesting their potential use in a larger range of patients. Clinical trials are clearly needed to substantiate the findings of this study while future studies characterizing the functional, metabolic, and molecular actions of SGLT2i in the vasculature might furnish insights into how these drugs eventually prevent the development of ISR-related events, overall providing the foundation and the rationale for a potential use of SGLT2i to prevent ISR in AMI patients with and, eventually, without diabetes.

## Supplementary Information

**Additional file 1: Supplementary Figure 1.** Risk of ISR-related MACE according to the use of different SGLT2i. Kaplan-Meier curves showing the risk of MACE in patients treated with either empagliflozin, canagliflozin, or dapagliflozin and compared with no-SGLT2i users, along with the relative values derived from Log Rank tests. No differences were observed between different drugs.

## Data Availability

The datasets used for this study is not publicly available due to the Italian legislation but is available from the corresponding author on reasonable request.

## Abbreviations

AMI: Acute myocardial infarction
BMI: Body mass index
CK-MB: Creatine phosphokinase-MB
CRP: C-reactive protein
eGFR: Estimated glomerular filtration rate
HbA1c: Glycated hemoglobin
HDL: High-density lipoprotein
ISR: In-stent restenosis
LDL: Low-density lipoprotein
LVEF: Left ventricular ejection fraction
MACE: Major adverse cardiovascular events
MLA: Minimum lumen area
MLD: Minimum lumen diameter
SGLT2i: Inhibitors of sodium-glucose transporter 2
T2DM: Type 2 diabetes mellitus
