# Supplementary material for: SGLT-2 inhibitors and in-stent restenosis-related events after acute myocardial infarction: an observational study in patients with type 2 diabetes
Source: BMC Med. 2023 Feb 24;21:71. doi: 10.1186/s12916-023-02781-2 (PMC9960194; doi:10.1186/s12916-023-02781-2)
Supplement: Supplementary file 1 — Additional file 1: Supplementary Figure 1. Risk of ISR-related MACE according to the use of different SGLT2i. Kaplan-Meier curves showing the risk of MACE in patients treated with either empagliflozin, canagliflozin, or dapagliflozin and compared with no-SGLT2i users, along with the relative values derived from Log Rank tests. No differences were observed between different drugs. [file 12916_2023_2781_MOESM1_ESM.pdf]

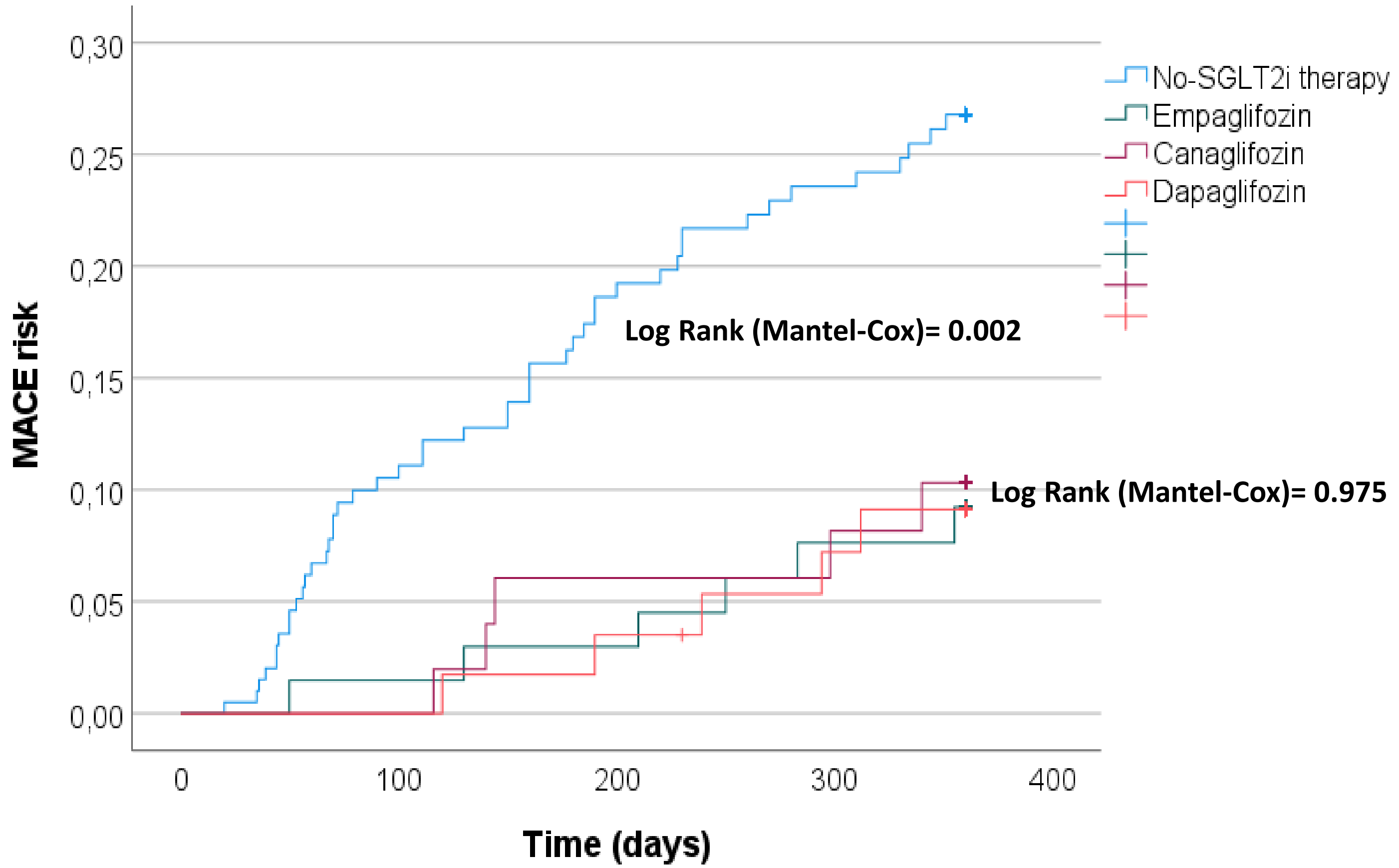

Supplementary Figure 1. Risk of ISR-related MACE according to the use of different SGLT2i. Kaplan-Meier curves showing the risk of MACE in patients treated with either empagliflozin, canagliflozin, or dapagliflozin and compared with no-SGLT2i users, along with the relative values derived from Log Rank tests. No differences were observed between different drugs.
